# Quantitative and Systems Pharmacology 3. Network-Based Identification of New Targets for Natural Products Enables Potential Uses in Aging-Associated Disorders

**DOI:** 10.3389/fphar.2017.00747

**Published:** 2017-10-18

**Authors:** Jiansong Fang, Li Gao, Huili Ma, Qihui Wu, Tian Wu, Jun Wu, Qi Wang, Feixiong Cheng

**Affiliations:** ^1^Institute of Clinical Pharmacology, Guangzhou University of Chinese Medicine, Guangzhou, China; ^2^Modern Research Center for Traditional Chinese Medicine, Shanxi University, Taiyuan, China; ^3^Department of Cancer Biology, Center for Cancer Systems Biology, Harvard Medical School, Dana-Farber Cancer Institute, Boston, MA, United States; ^4^Center for Complex Networks Research, Northeastern University, Boston, MA, United States

**Keywords:** quantitative and systems pharmacology, natural products, target identification, aging, network-based

## Abstract

Aging that refers the accumulation of genetic and physiology changes in cells and tissues over a lifetime has been shown a high risk of developing various complex diseases, such as neurodegenerative disease, cardiovascular disease and cancer. Over the past several decades, natural products have been demonstrated as anti-aging interveners via extending lifespan and preventing aging-associated disorders. In this study, we developed an integrated systems pharmacology infrastructure to uncover new indications for aging-associated disorders by natural products. Specifically, we incorporated 411 high-quality aging-associated human genes or human-orthologous genes from *mus musculus* (MM), *saccharomyces cerevisiae* (SC), c*aenorhabditis elegans* (CE), and *drosophila melanogaster* (DM). We constructed a global drug-target network of natural products by integrating both experimental and computationally predicted drug-target interactions (DTI). We further built the statistical network models for identification of new anti-aging indications of natural products through integration of the curated aging-associated genes and drug-target network of natural products. High accuracy was achieved on the network models. We showcased several network-predicted anti-aging indications of four typical natural products (caffeic acid, metformin, myricetin, and resveratrol) with new mechanism-of-actions. In summary, this study offers a powerful systems pharmacology infrastructure to identify natural products for treatment of aging-associated disorders.

## Introduction

Aging is a complex biological process accompanied by accumulation of degenerative damages as well as the decline of various physiological function, leading to the death of an organism ultimately (Fontana et al., 2010; Lopez-Otin et al., 2013; Vaiserman et al., 2016). As an inevitable outcome of life, aging is a primary risk factor for various complex diseases, including cancer, cardiovascular diseases, and neurodegenerative disease (Kaeberlein et al., 2015; Vaiserman and Marotta, 2016). Thus, development of novel agents for delaying or preventing aging-associated disorders plays essential roles during drug discovery and development.

Natural products have been demonstrated preclinical or clinical efficiency for developing anti-aging interveners with few side effects (Ding et al., 2017). Over the past few decades, several natural products have been reported as anti-aging agents to extend lifespan and prevent aging-associated diseases in various organism and animal models (Pan et al., 2012; Correa et al., 2016). Currently, over 300,000 natural products have been available for drug discovery and development (Banerjee et al., 2015). Among of them, 547 natural products and derivatives have been approved by U.S. Food and Drug Administration (FDA) for treating or preventing various disorders by the end of 2013 (Patridge et al., 2016). There is pressing need of novel approaches or tools for systematic identification of natural products with novel pharmacotherapeutic mechanism-of-action for treatment of aging-associated disorders.

Traditional drug target identification includes ligand-based and structure-based approaches, such as machine learning and molecular docking (Fang et al., 2013, 2017b). However, machine learning is limited by high quality of negative samples as well as overfitting issues on small training sets, while molecular docking is constrained by lack of available crystallographic three-dimensional (3D) structures of proteins. To overcome the pitfalls of traditional approaches, several network-based approaches for prediction of drug-target interaction (DTI) have been proposed recently (Cheng et al., 2012a,b; Wu et al., 2016, 2017). These approaches have showed a great promise in drug discovery and development, since they do not rely on either 3D structures of proteins or negative DTIs.

Quantitative and systems pharmacology refers to a multidisciplinary approach for the emerging development of efficacious drugs with novel mechanisms via integration of experimental assays and computational strategies (Vicini and van der Graaf, 2013; Fang et al., 2017b,c). In the past decade, systems pharmacology-based approaches have demonstrated advance in drug discovery and development (Lu et al., 2015; Cheng et al., 2016; Fang et al., 2017a). For example, a recent study has reported a systems pharmacology approach for identifying new anticancer indications via integrating drug-gene signatures from the connectivity map into the cancer driver genes derived from tumor-normal matched whole-exome sequencing data (Cheng et al., 2016). They identified several new anticancer indications of resveratrol with new molecular mechanisms. Recently, the same group further proposed a system pharmacology approach that facilitated to identify new anticancer indications of natural products through integration of known DTI network into significantly mutated genes in cancer (Fang et al., 2017a). The high-confidence anticancer indications were identified computationally and further validated by various literatures on four natural products, including resveratrol, quercetin, fisetin, and genistein. They showed that integration of the computationally predicted DTIs could significantly enhance the success rate of identifying new anticancer indications of natural products via reducing the incompleteness of known drug-target networks. The aforementioned examples have shed light on the systems pharmacology-based approaches for drug discovery through exploiting the polypharmacology of natural products with pleiotropic effects for treatment of various complex diseases (Fang et al., 2017b).

**Figure 1:**
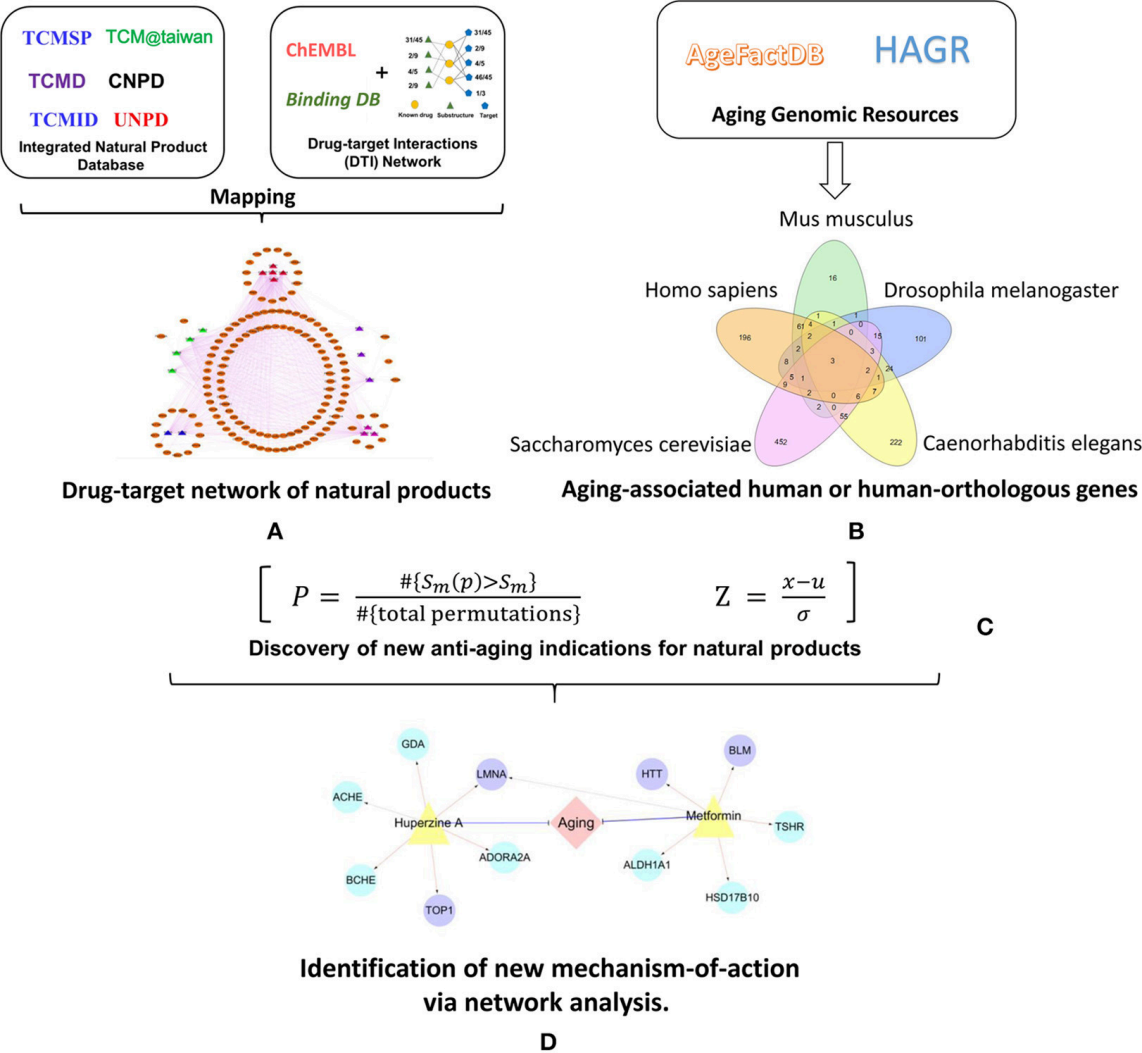
Schematic diagram of the systems pharmacology infrastructure for identification of aging-associated indications by natural products. **(A)** Construction of drug-target network of natural products. **(B)** Manual curation of aging-associated genes. **(C)** Discovery of new anti-aging indications for natural products via network-based prediction. **(D)** Identification of new anti-aging mechanism-of-action via network analysis.

In this study, we further proposed an integrated systems pharmacology framework (Figure 1) to identify new targets of natural products for potential treatment of aging-associated disorders. Specifically, we manually collected high-quality aging-associated human genes or human-orthologous genes covering four species: *caenorhabditis elegans* (CE), *drosophila melanogaster* (DM), *mus musculus* (MM), and *saccharomyces cerevisiae* (SC). We reconstructed a global DTI network of natural products by integrating both experimentally reported and computationally predicted DTIs from our previous predictive network models (Wu et al., 2016; Fang et al., 2017c). Finally, we built the statistical network models with high accuracy to prioritize new anti-aging indications of natural products through integration of the curated aging-associated genes and drug-target network of natural products. We computationally identified anti-aging indications of multiple natural products with novel molecular mechanisms, providing potential promising candidates for further treatment of aging-associated diseases. Taken together, this study offers a powerful systems pharmacology infrastructure for identification of natural products with new mechanism-of-action for potential treatment of aging-associated disorders.

## Materials and methods

### Manual curation of aging-associated genes

Aging-associated genes (AAGs) were collected from two comprehensive databases: the JenAge Ageing Factor Database (AgeFactDB) (Huhne et al., 2014) and Human Ageing Genomic Resources database (HAGR) (Tacutu et al., 2013). AgeFactDB collects and integrates aging phenotype data with both experimental and computational evidence, while HAGR only contains AAGs from experiments. In this study, we only extracted AAGs from AgeFactDB and HAGR with well-known experimental evidences across five organisms: *Homo sapiens* (HS), CE, DM, MM, and SC. After removing the duplicated genes between two databases, we obtained 309 (HS), 194 (DM), 1,012 (SC), 1,149 (CE), and 143 (MM) AAGs, respectively. We then obtained high-quality human-orthologous AAGs via mapping human-orthologous genes across four species (CE, DM, MM, and SC) from ensemble database (http://www.ensembl.org/index.html). Finally, 169 (DM), 555 (SC), 331 (CE), and 96 (MM) human-orthologous AAGs were collected (Table S1).

### Construction of a known drug-target network of natural products

We firstly integrated comprehensive natural products from six publically available natural product-related data sources: traditional Chinese medicine database (TCMDb) (He et al., 2001), Chinese natural product database (CNPD) (Shen et al., 2003), traditional Chinese medicine integrated database (TCMID) (Xue et al., 2013), traditional Chinese medicine systems pharmacology (TCMSP) (Ru et al., 2014), traditional Chinese medicine database@Taiwan (TCM@Taiwan) (Chen, 2011), and universal natural product database (UNPD) (Gu et al., 2013). For each data source, we converted its initial structure format (e.g., mol2) into unified SDF format. Secondly, we merged the unified SDF files from the six data sources into single SDF file, and removed the duplicated natural products according to InChIKey by Open Babel (v2.3.2) (O'Boyle et al., 2011). Finally, 259,547 unique natural products were collected. The details are provided in our previous study (Fang et al., 2017a,c).

To construct a global drug-target network of natural products, we pooled DTIs from two commonly used databases: ChEMBL (v21) (Bento et al., 2014) and BindingDB (v19, accessed in June 2016) (Gilson et al., 2016). All chemical structures were carefully standardized via removing salt ions and standardizing dative bonds using Open Babel toolkit (v2.3.2). We further filtered DTIs with the following five criteria: (i) Ki, Kd, IC_50_, or EC_50_ ≤ 10 μM; (ii) the target organism should be homo sapiens; (iii) the target has a unique UniProt accession number; (iv) compound can be transformed to canonical SMILES format; and (v) compound has at least one carbon atom. Subsequently, we extracted experimentally validated DTIs for 2,349 natural products after mapping 259,547 unique natural products into the global DTIs using the “InChIKey.”

### Prediction of new drug-target interactions of natural products

In a recent study, we have developed predictive network models to predict targets of natural products via a balanced substructure-drug-target network-based inference (bSDTNBI) (Fang et al., 2017c; Wu et al., 2017) approach. The bSDTNBI utilizes resource-diffusion processes to prioritize potential targets for both known drugs and new chemical entities (NCEs) via substructure-drug-target network (Wu et al., 2016). The substructure-drug (or NCE)-target network was built via integrating the known DTI network, drug-substructure associations and NCE-substructure associations. Two parameters were introduced to balance the initial resource allocation of different node types (α) and the weighted values of different edge types (β), respectively. The third parameter γ was imported to balance the influence of hub nodes in resource-diffusion processes. The fourth parameter *k* denotes for the number of resource diffusion processes. Herein, four parameters (α = β = 0.1, γ = −0.5, and *k* = 2) in bSDTNBI were adopted based on our previous study (Wu et al., 2016). Here, the predictive model based on KR molecular fingerprint (bSDTNBI_KR) with the best performance was used to predict the new targets of natural products and top 20 predicted candidates were used (Wu et al., 2016, 2017).

### Identification of new anti-aging indications for natural products

Here, we further proposed an integrated statistical network model to prioritize new anti-aging indications of natural products by incorporating DTI network of natural products and the manually curated AAGs. We asserted that a natural product with polypharmacological profiles exhibits a high possibility to treat an aging-associated disorder if its targets are more likely to be aging-associated proteins (AAPs). Then we utilized a permutation testing to estimate the statistical significance of a natural product to be prioritized for anti-aging indications. The null hypothesis asserts that targets of a natural product randomly locate at AAPs across the human proteome. The permutation testing was performed as below:

(1) $$\begin{array}{r} P\;=\;\frac{\#\;\left\{S_{m}\left(p\right)>S_{m}\right\}}{\#\;\left\{\text{total permutations}\right\}} \end{array}$$

A nominal *P* was computed for each natural product by counting the number of observed AAPs greater [*S*_*m*_ *(p)*] than the permutations (*Sm*). Here we repeated 100,000 permutations by randomly selecting 441 proteins (the same number of AAPs) from protein products at the genome-wide scale, 20,462 human protein-coding genes from the National Center for Biotechnology Information (NCBI) database (Coordinators, 2017; Table S2). Subsequently, the nominal *P*-values from the permutation tests were corrected as adjusted *P*-values (q) based on Benjamini-Hochberg approach (Benjamini and Hochberg, 1995) using R package (v3.01). In addition, a Z-score was calculated for each natural product to be prioritized for anti-aging indications during permutation testing:

(2) $$\begin{array}{r} Z\;=\;\frac{x-\mu }{\sigma } \end{array}$$

where *x* is the real number of AAPs targeted by a given natural product, μ is the mean number of AAPs targeted by a given natural product during 100,000 permutations, and σ is the standard deviation.

### Network and statistical analysis

The statistical analysis in this study was carried out using the Python (v3.2, http://www.python.org/) and R platforms (v3.01, http://www.r-project.org/). Networks were visualized by Cytoscape (v3.2.0, http://www.cytoscape.org/).

## Results

### A catalogue of aging-associated genes

**Figure 2:**
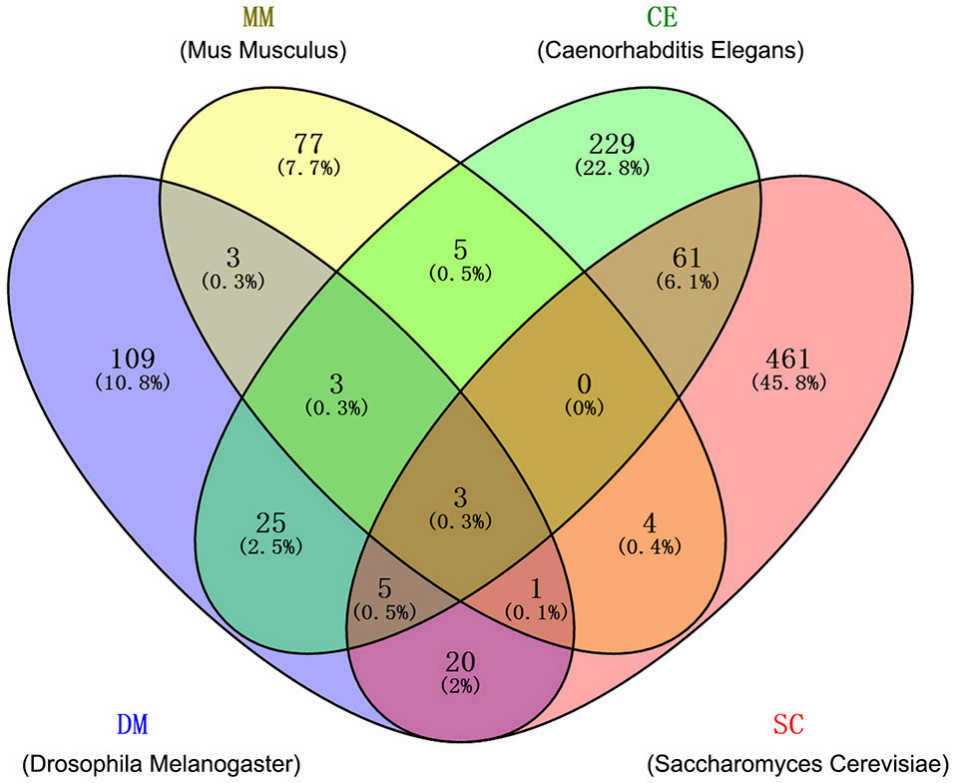
Overlaps among four gene sets of human-orthologous aging-associated genes (AAGs) from 4 non-human organisms: *Caenorhabditis elegans* (CE), *Drosophila melanogaster* (DM), *mus musculus* (MM), and *saccharomyces cerevisiae* (SC). The detailed AAGs are provided in Table S1.

We collected the high-quality human-orthologous AAGs from four species: CE, DM, MM, and SC. In total, 1,006 human-orthologous AAGs identified in at least one species with literature-reported experimental evidences were collected after removing the duplicated AAGs (Figure 2). Among 1,006 genes, 130 human-orthologous AAGs are reported in at least two non-human organisms (CE, DM, MM and SC) simultaneously. Meanwhile, 12 human-orthologous AAGs (e.g., *AKT1, CAT, GABARAP, MAPK8, MAPK9, MAPK10, MTOR, PRDX1, PRDX2, RPS6KB1, SIRT1*, and *SOD2*) were included in at least three non-human organisms. To improve the quality of gene set, we only selected the 130 human-orthologous AAGs identified in at least two non-human organisms. We found that 130 human-orthologous AAGs are significantly enriched with 309 human AAGs (28 overlapping genes, *P* = 1.7 × 10^−24^, Fisher's exact test). Finally, we pooled 130 human-orthologous AAGs and 309 human AAGs and generated 411 AAGs (Table S3) for building the statistical network models.

### Reconstruction of anti-aging drug-target network for natural products

**Figure 3:**
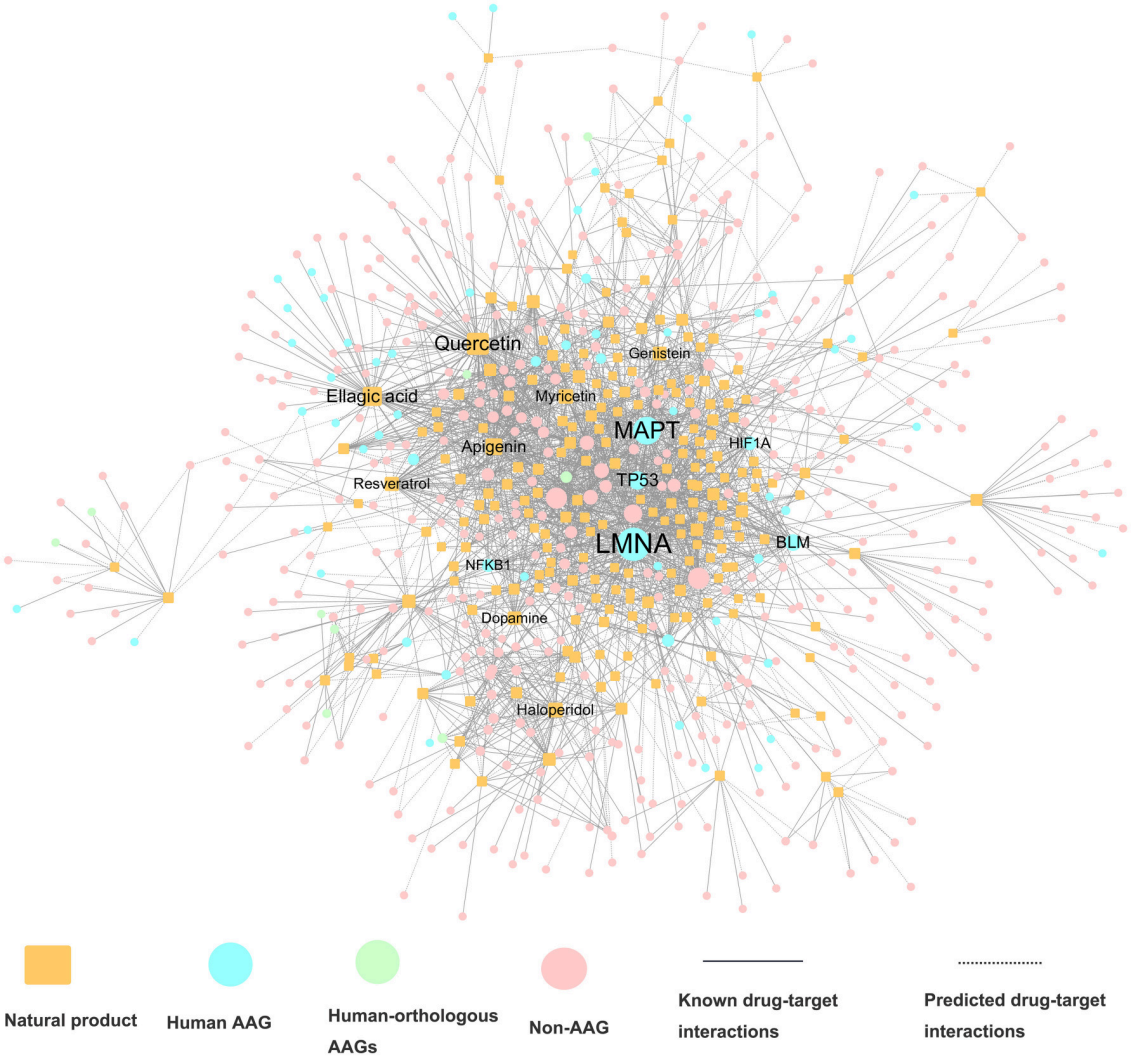
A bipartite drug–target interaction network for FDA-approved or clinically investigational natural products. This network contains 2,408 interactions connecting 224 natural products to 494 target proteins, including proteins encoded by 70 aging-associated genes (AAGs) and 424 non-AAGs. The label font size and node size are proportional to degree (connectivity).

We constructed a global drug-target network of natural products by integrating 7,314 high-quality experimental DTIs as well as 11,940 new computational predicted DTIs as described in our recent study (Fang et al., 2017c). The global DTI network (Table 1) was consisted of 17,223 DTIs connecting 2,349 unique natural products and 732 targets. The average experimental target degree (connectivity) of a natural product is 2.97, which is significantly stronger than the average degree 2.22 of non-natural product drugs in DrugBank database (*P* = 6.81 × 10^−72^, one-side Wilcoxon test). The detailed DTI pairs are provided in Table S4. We further built a specific drug-target network by focusing on FDA-approved or clinically investigational natural products (Table S4). Figure 3 displays a bipartite drug-target network of 2,408 DTIs connecting 224 FDA-approved or clinically investigational natural products and 494 targets encoded by 70 AAGs and 424 non-AAGs. Network analysis shows that the average connectivity of experimentally known targets for each natural product in this network is 6.26, which is significantly stronger than that (average degree = 2.22) of non-natural products drugs in DrugBank (*P* = 4.34 × 10^−50^, one-side Wilcoxon test, Table S5). Among 224 FDA-approved or clinically investigational natural products, eight natural products have connectivity (*K*) > 25: quercetin (*K* = 73), ellagic acid (*K* = 56), apigenin (*K* = 43), haloperidol (*K* = 32), myricetin (*K* = 32), resveratrol (*K* = 30), genistein (*K* = 26), and dopamine (*K* = 25). Meanwhile, among 70 targets encoded by AAGs, 6 are targeted by over 15 natural products (D): LMNA (*D* = 79), MAPT (*D* = 33), BLM (*D* = 22), HIF1A (*D* = 22), TP53 (*D* = 20), and NFKB1 (*D* = 16), based on current available experimental data. The targets encoded by these AAGs play essential roles in aging-associated diseases. For example, products encoded by *LMNA* are primarily lamin A and C. Alterations in lamin A and C were reported to accelerate physiological aging via nuclear envelope budding (Li Y. et al., 2016). A recent study also showed that nuclear factor-kappa B (NF-kB) inhibition could delay the onset of aging symptoms in mice via reducing DNA damage (Tilstra et al., 2012).

**Table 1:** The statistics of global drug-target interactions (DTI) network and local DTI network for natural products.

**Data set**	**N_D_**	**N_T_(N_AT_)**	**N_DTI_**	**Sparsity (%)**
Global DTI network	2,349	732 (101)	17,223	1.00
Local DTI network	224	494 (70)	2408	2.17

*Local DTI network: a specific drug-target network by focusing on FDA-approved or clinically investigational natural products, N_D_, the number of natural products; N_T_, the number of targets; N_AT_, the number of aging-associated targets; N_DTI_, the number of DTIs; Sparsity, the ratio of N_DTI_ to the number of all possible DTIs*.

### Chemical diversity analysis of natural products targeting aging-associated proteins

**Figure 4:**
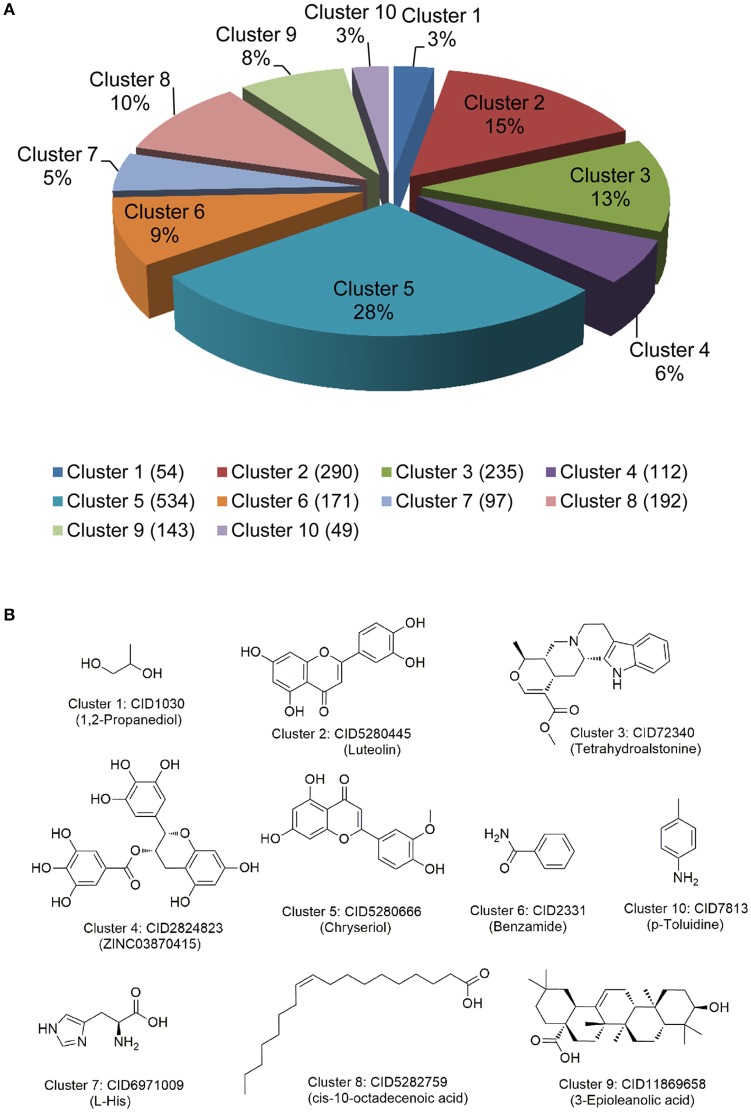
Chemical diversity analysis of natural products targeting aging-associated proteins. **(A)** Chemical structure clustering of 1,877 natural products via FCFP_6 fingerprint; **(B)** The representative structures of 10 cluster centers during chemical structural clustering analysis.

We extracted 1,877 natural products targeting AAP via mapping 411 high-quality human or human-orthologous AAGs into the global drug-target network of natural products. Clustering analysis was performed to examine chemical scaffolds of 1,877 natural products by measuring the root-men-square value of the Tanimoto distance based on FCFP_6 fingerprint implemented in Discovery Studio 4.0 (version 4.0, Accelrys Inc.). The 1,887 natural products are clustered into 10 groups with cluster centers: 1,2-propanediol, luteolin, tetrahydroalstoine, ZINC03870415, chryseriol, benzamide, p-toluidine, L-His, cis-10-octadecenoic acid, and 3-epioleanolic acid, respectively (Figure 4A). The structures of each cluster center are shown in Figure 4B. Among them, cluster 5 (Cluster center: Chryseriol) and cluster 2 (Cluster center: Luteolin) are grouped as flavonoids, with the largest number of natural products. The structures in cluster 3 and cluster 9 are represented as alkaloids, while the structures in cluster 8 are represented as unsaturated aliphatic hydrocarbon or unsaturated fatty acid. Overall, 1887 natural products share diverse chemical scaffolds (Figure 4), providing a valuable resource for systems pharmacology-based anti-aging drug discovery.

### Mechanism-of-action of anti-aging indications by natural products

To investigate the anti-aging mechanism-of-action (MOA) of natural products, we performed KEGG pathway, molecular function, and biological process enrichment analysis using ClueGO (Bindea et al., 2009). Here, we focused on 54 AAPs with connectivity larger than 10 in the global drug-target network of natural products (Table S6). Figure S1 showed that 54 anti-aging targets are significantly enriched in several aging-associated pathways: longevity regulating pathway (adjusted-*P* = 1.9 × 10^−5^), MAPK signaling pathway (adjusted-*P* = 1.6 × 10^−5^), ERBB signaling pathway (adjusted-*P* = 7.8 × 10^−7^), estrogen signaling pathway (adjusted-*P* = 3.3 × 10^−4^), and insulin signaling pathway (adjusted-*P* = 2.1 × 10^−3^) (Hall et al., 2017). Similar trends were observed for molecular function and biological process enrichment analyses (Table S7). To further showcase the aging-associated mechanisms, we selected four typical natural products: caffeic acid, hesperetin, myricetin and resveratrol.

#### Caffeic acid

**Figure 5:**
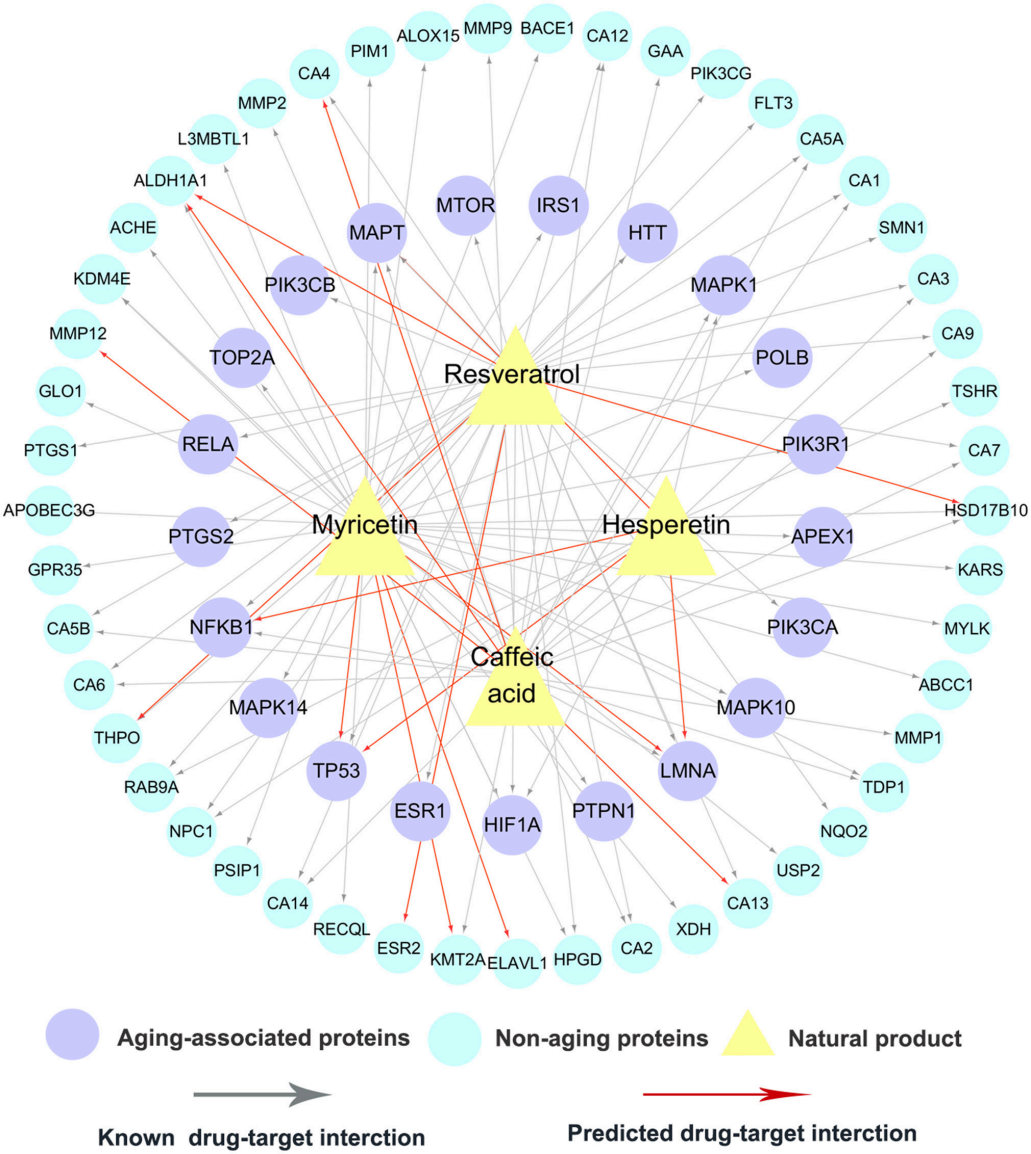
A bipartite drug-target network for 4 typical natural products. This network includes 90 experimentally validated and 16 computationally predicted drug-target interactions connecting 4 natural products (caffeic acid, hesperetin, myricetin and resveratrol) and 70 targets (21 aging-associated proteins and 49 non-aging proteins).

Caffeic acid is a natural phenol found in fruits, tea and wine (Magnani et al., 2014), with a wide range of aging-associated pharmacological activities, such as antioxidant (Deshmukh et al., 2016), anti-inflammatory (da Cunha et al., 2004), and neuroprotective (Pereira et al., 2006). For example, caffeic acid phenethylester (CAPE) was reported to extend lifespan in CE via regulation of the insulin-like DAF-16 signaling pathway (Havermann et al., 2014). The detailed molecular mechanisms of anti-aging effects by caffeic acid remain unclear. Figure 5 shows that caffeic acid interacts with 5 AAPs (LMNA, MAPT, NFKB1, PTPN1 and MAPK1) and 22 non-AAPs, consisting of 23 experimentally validated and 4 computationally predicted ones. Protein tyrosine phosphatase 1B (PTP1B), encoded by *PTPN1*, is a potential target for treatment of type-2 diabetes (Gonzalez-Rodriguez et al., 2012) and Alzheimer's disease (Vieira et al., 2017). A recent study showed that caffeic acid is a moderate inhibitor of PTP1B with an IC_50_ value of 3.06 μM (He et al., 2009).

#### Hesperetin

Hesperetin is flavanone abundant in citrus fruits with a wide range of biological activities. Recent studies revealed the potential antioxidant, neuroprotective, and anti-inflammatory properties (Parhiz et al., 2015; Miler et al., 2016), by hesperetin. Furthermore, a recent clinical trial (NCT02095873) has reported that hesperetin in combination with trans-resveratrol can prevent and alleviate early-stage of aging-associated disorders (Xue et al., 2016). Network analysis reveals that hesperetin binds with 9 targets (6 AAPs and 3 non-AAPs), including 4 computationally predicted targets and 5 experimentally reported ones. Interestingly, 4 predicted anti-aging targets (MAPT, LMNA, TP53, and NFKB1) suggest potential underlying anti-aging mechanisms by hesperetin. For example, a previous study revealed that hesperetin modulated the aging-associated NF-κB pathway in the kidney of rats (Kim et al., 2006).

#### Myricetin

Myricetin, a common plant-derived flavonoid, displays several pharmacological activities against aging-associated indications, such as anti-aging (Aliper et al., 2016), antioxidant (Wang et al., 2010), anti-inflammatory (Lee et al., 2007), and immunomodulatory (Fu et al., 2013) effects. Figure 5 shows that myricetin binds with 11 AAPs and 25 non-AAPs, consisting of 4 computationally predicted targets (TP53, LMNA, ELAVL1, and KMT2A) and 32 experimentally reported ones. A recent study has suggested that myricetin can extend lifespan in *Caenorhabditis elegans* via modulating aging-related transcription factors (Buchter et al., 2013).

#### Resveratrol

Resveratrol, a non-flavonoid polyphenol abundant in the skin of grapes, displays a broad spectrum of anti-aging effects (Gines et al., 2017). Currently, over 20 clinical trials (http://clinicaltrials.gov/) are being conducted or completed to treat aging or aging-associated disorders by resveratrol, such as anti-aging (NCT02523274 and NCT02909699), aging-associated macular degeneration (NCT02625376), and Alzheimer's disease (NCT01504854). Figure 5 indicates that resveratrol interacts with 12 AAPs: ESR1, HIF1A, HTT, LMNA, MAPT, MTOR, NFKB1, PIK3CA, PIK3CB, PTGS2, RELA, and TP53, suggesting new potential anti-aging mechanisms of resveratrol. For example, two AAPs: estrogen receptor alpha (ER-alpha) and cyclooxygenase-2 (COX-2), play crucial role on the pathogenesis of several aging-associated diseases, such as Alzheimer's disease and osteoporosis (Kermath et al., 2014; Kim et al., 2016). Resveratrol was reported to bind to ER-alpha with a Ki value of 0.78 μM (de Medina et al., 2005) and inhibit COX-2 with an IC_50_ value of 0.99 μM (Kang et al., 2009).

Taken together, aforementioned examples demonstrated that network analysis could assist to identify new potential anti-aging mechanisms of natural products. Systems pharmacology-based integration of drug-target networks and known AAPs would enable to identify new natural products for treatment of aging-associated diseases.

### Discovery of potential anti-aging indications for natural products with novel mechanism-of-action

We further built statistical network models for comprehensive identification of new anti-aging indications of natural products through integrating both experimentally reported and computationally predicted drug-target network into the curated APPs (see section Materials and Methods). Here, we focused on 224 FDA-approved or clinical investigational natural products annotated in DrugBank database (Law et al., 2014). Table 2 summarizes number of the predicted anti-aging indications for the experimentally reported drug-target network only and the pooled data from both experimentally reported and computationally predicted drug-network, respectively. We only identified 56 natural products with significantly predicted anti-aging indications (*q* < 0.05) using the experimentally reported drug-target network, while we identified 143 natural products with significantly predicted anti-aging indications (*q* < 0.05) via integration of both experimentally reported and computationally predicted drug-target networks (Table S8). Interestingly, among 143 natural products, 92 natural products cannot be identified to have significant anti-aging indications using experimentally reported drug-target network only, including some well-known anti-aging natural products (e.g., metformin, vitamin E, and huperzine A). We systematically retrieved previously anti-aging reported data from PubMed for 73 FDA-approved natural products out of 143 ones. The detailed experimental evidences are provided in Table S9. Then we found 23 natural products [with a success rate of 31.5% (23/73)] with reported experimental data. This suggests a reliable accuracy of our proposed network model. The remaining 50 natural products without experimental data provide potential anti-aging candidates that deserve to be validated by various experimental assays in the future.

**Table 2:** Summary of the newly predicted anti-aging indications of natural products based on the experimentally reported drug-target network only (ExpNet) and the combination of the experimentally reported and computationally predicted (ExpNet&ComNet) drug-target networks, respectively.

**Data source**	**Number of DTIs (number of targets, number of drugs)**	**# N_saI_ (*q* < 0.05)**	**# N_saI_ (*q* < 1/10^−5^)**
ExpNet	1,163 (361,113)	56	28
ExpNet&ComNet	2,408 (494, 224)	143	87

*N_saI_ denotes to the number of natural products with significantly predicted anti-aging indication*.

In summary, we showed that integration of computationally predicted drug-target network could improve the chance to identify new anti-aging indications of natural products via increasing completeness of current drug-target network. We next chose three typical natural products (metformin, vitamin E, and huperzine A) as case studies to illustrate the predicted anti-aging indications with new mechanism-of-actions.

#### Metformin

**Figure 6:**
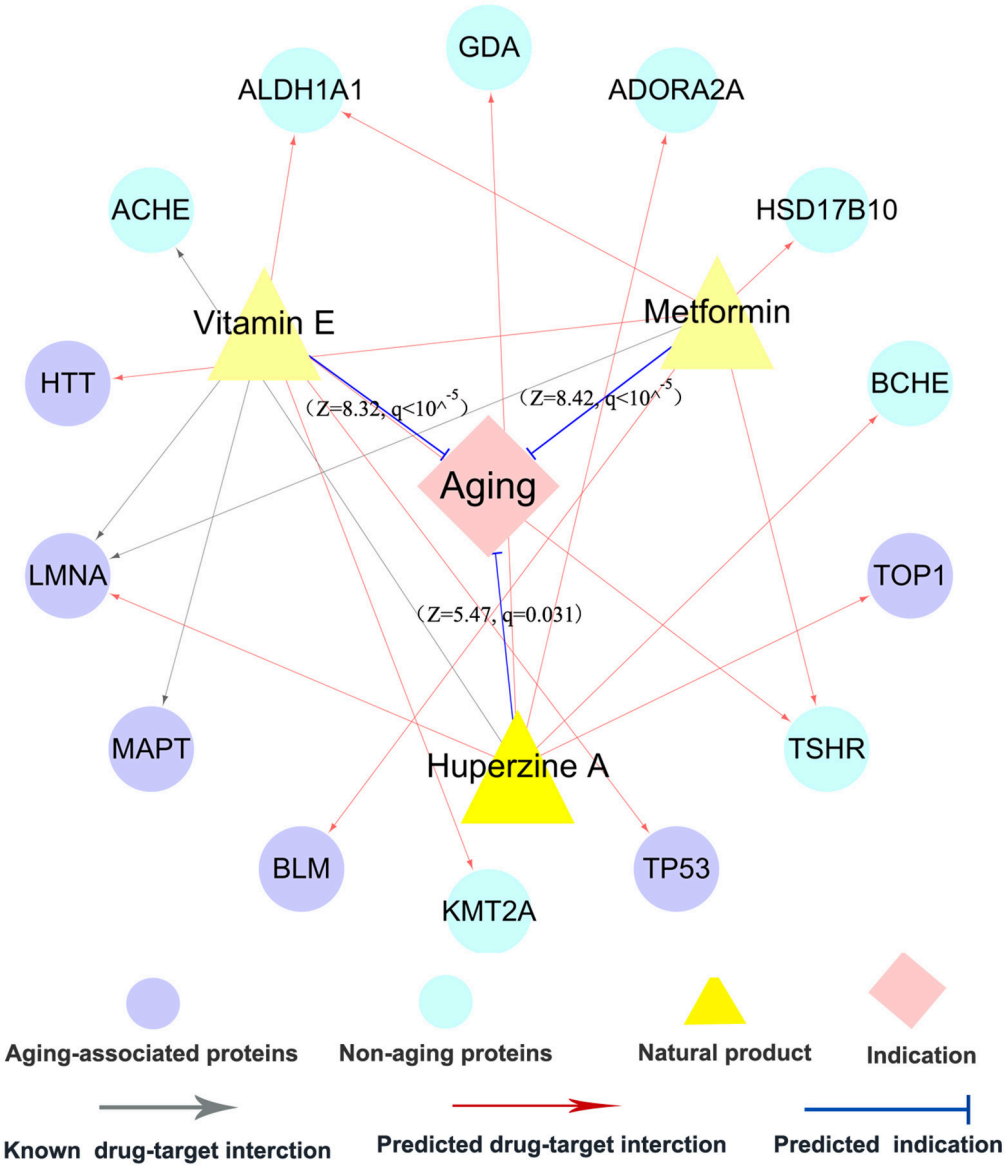
A discovered anti-aging drug-target network for 3 typical natural products. This network displays the predicted anti-aging indications as well as the known and predicted drug targets for three typical natural products: metformin, vitamin E, and huperzine A. The thickness of blue line between natural products and anti-aging indication is proportional to the predicted Z-score (Equation 2, see section Materials and Methods).

Metformin, originating from *Galega officinalis*, is a biguanide drug widely used in clinical practice for treating type-2 diabetes. Nowadays, metformin is currently being tested as an anti-aging drug in several clinical trials, such as NCT02432287 and NCT02308228 (Barzilai et al., 2016). Figure 6 shows that metformin binds with 3 AAPs (BLM, HTT, and LMNA) and 3 non-APPs. In our network model, metformin was predicted to have significant anti-aging indication (*Z* = 8.42, *q* < 10^−5^) via integration of one experimentally validated target and five predicted ones. There is no significant anti-aging indication for metformin based on experimentally validated DTI only. Previous studies have shown that metformin extended lifespan in several model organisms (Anisimov, 2013; Cabreiro et al., 2013; Martin-Montalvo et al., 2013). Figure 6 shows several potential anti-aging mechanisms of metformin, including inhibition of the inflammatory pathway, activation of AMP-activated kinase (AMPK), and inducing autophagy (Moiseeva et al., 2013; Foretz et al., 2014; Song et al., 2015).

#### Vitamin E

Vitamin E, the most potent antioxidant, protects cells from damage related to oxidative stress (La Fata et al., 2014). Vitamin E supplementation has been reported to delay or prevent aging and inflammatory aging-associated diseases via prolonging the life span in several model organisms (Navarro et al., 2005; Mocchegiani et al., 2014). However, mechanism-of-action of anti-aging effects by vitamin E remains unclear. Figure 6 shows that vitamin E interacts with 2 known and 4 predicted targets, consisted of 3 AAPs (TP53, LMNA, and MAPT) as well as 3 non-AAPs. In our network model, vitamin E was predicted to show potential for anti-aging indication (*Z* = 8.32, *q* < 10^−5^) based on the pooled data of experimentally validated and computationally predicted DTIs. However, there is no significance based on experimentally validated DTIs only. Among 4 predicted targets, TP53, a well-known AAP, regulates cell cycle progression, apoptosis, and cellular senescence. A recent study reported that vitamin E significantly down-regulated *TP53* expression in senescent cells, indicating a potential anti-aging mechanism of vitamin E (Durani et al., 2015).

#### Huperzine A

Huperzine A (HupA), a natural acetylcholinesterase (ACHE) inhibitor derived from Huperzia serrate, is a licensed anti-Alzheimer drug in China (Qian and Ke, 2014). HupA was reported to show various anti-inflammatory, neuroprotective, and anti-aging properties (Ruan et al., 2013; Damar et al., 2016). Figure 6 reveals that HupA binds with one experimentally reported target (ACHE) and 5 computationally predicted targets (BCHE, GDA, LMNA, TOP1, and ADORA2A). Among five predicted targets, LMNA and TOP1 are experimentally reported AAPs (Li Y. et al., 2016). Here, HupA was predicted to have significant anti-aging indication (*Z* = 5.47, *q* = 0.031) via the integration of both experimentally reported and computationally predicted targets, while no significance using the experimentally reported targets alone. Oxidative stress accelerates the chronic inflammatory process during aging and aging-associated diseases (Cannizzo et al., 2011). A previous study showed that HupA alleviated oxidative stress-induced inflammatory damage in aging rat (Ruan et al., 2014).

Put together, we have suggested that our network model provided a useful tool for systematic identification of natural products for treatment of aging-associated disorders with novel molecular mechanisms. Some newly predicted anti-aging indications of natural products and the according mechanisms are suggested to be experimentally validated before clinical uses, which we hope to be promoted by findings shown here.

## Discussion

Natural products are valuable pharmaceutical wealth and show great promise for developing anti-aging agents (Ding et al., 2017). In this study, we developed an integrated systems pharmacology infrastructure to identify new targets of natural products for treatment of aging-associated diseases. This computational infrastructure is consisted of three key components: (i) reconstructing DTI networks of natural products via integrating known and computationally predicted DTIs; (ii) curation of high-quality aging-associated human or orthologous genes from various aging-related bioinformatics sources; (iii) building statistical network models to prioritize new aging-associated indications of natural products through integrating data from aforementioned two steps. Overall, this framework has several advantages. First, we found that assembling computationally predicted drug-target network could identify more significant anti-aging indications for natural products by increasing completeness of currently known drug-target network. Second, our systems pharmacology-based approach is independent of three-dimensional (3D) structure of targets, which can be applied in human targets without known 3D structures (e.g., membrane proteins).

There are still several potential limitations in the current systems pharmacology model. First, antagonistic or agonistic effects of drug–target pairs have not been considered. Drug-induced gene expression database, such as the Connectivity Map (CMap), has provided specific biological functions (upregulation or downregulation) (Lamb et al., 2006). Integration of large-scale gene expression profiles of natural products may help improve performance of our network model (Cheng et al., 2016). In addition, current approach can only predict the potential aging-associated indications of natural products targeting known or predicted AAPs. Integrating systems biology resources may assist on identifying the growing potential AAPs by indirectly targeting their neighbors in the human protein-protein interaction network, gene regulatory network, or biological pathways (Li et al., 2014; Li J. et al., 2016). Finally, we only focused on three well-known natural products (metformin, vitamin E, and huperzine A) with more available literature-reported data for validation. Further *in vitro* or *in vivo* experimental assays should be performed to validate the predicted DTIs and anti-aging effects of natural products before preclinical and clinical studies.

## Author contributions

JF and FC conceived the project. FC and QWa provided supervision. JF and LG performed the research. JF, HM, QWu, TW, JW, and FC analyzed the data. JF and FC wrote the article.

### Conflict of interest statement

The authors declare that the research was conducted in the absence of any commercial or financial relationships that could be construed as a potential conflict of interest.

## Supplementary material

Figure S1 Pathway, biological, functional enrichment analyses of 54 aging-associated targets in global drug-target network of natural products. **(A)** KEGG pathway enrichment analysis; **(B)** Biological process enrichment analysis; **(C)** Molecular function enrichment analysis.

Table S1 List of 1,460 curated aging-associated genes (AAGs) across five organisms.

Table S2 List of 20,462 human protein-coding genes from the NCBI database.

Table S3 List of 411 essential human or human-orthologous aging-associated genes (AAGs).

Table S4 The detailed information of two drug-target interaction (DTI) networks (a global DTI network and a specific DTI network for FDA-approved or clinically investigational natural products).

Table S5 The detailed information of known experimental targets for both natural products and non-natural products collected from DrugBank for Wilcoxon test.

Table S6 List of 54 aging-associated proteins of which degree (connectivity) were higher than 10.

Table S7 The detailed information for ClueGO analysis.

Table S8 List of predicted indications of natural products based on experimental drug-target network only and the pooled drug-target network from both computationally predicted and experimentally validated drug-target interactions.

Table S9 The anti-aging reported data in PubMed for 73 FDA-approved natural products.
