# Supplementary figures and images for: Quantitative and Systems Pharmacology 3. Network-Based Identification of New Targets for Natural Products Enables Potential Uses in Aging-Associated Disorders
Source: Front Pharmacol. 2017 Oct 18;8:747. doi: 10.3389/fphar.2017.00747 (PMC5651538; doi:10.3389/fphar.2017.00747)

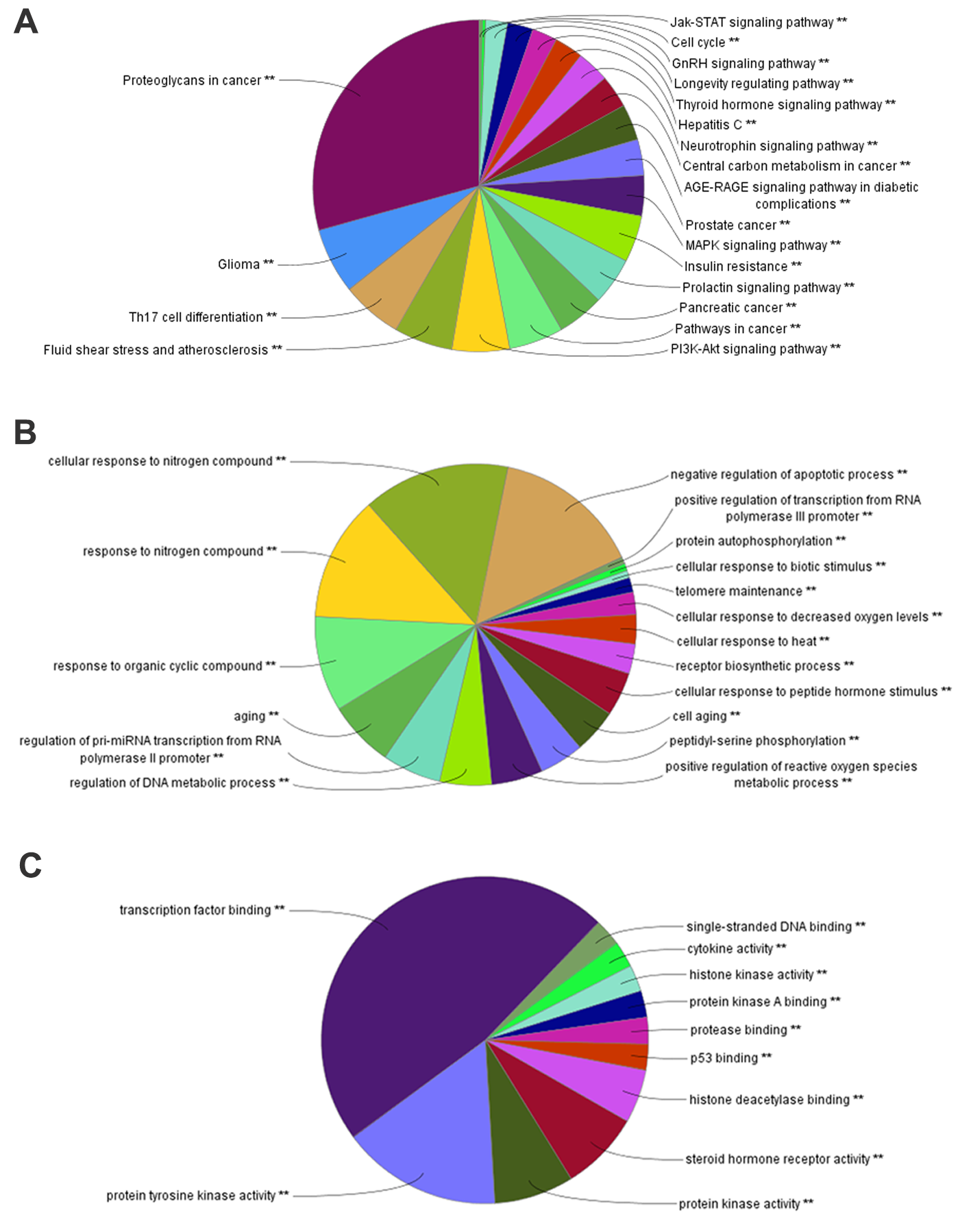

Supplement: Figure S1 — Pathway, biological, functional enrichment analyses of 54 aging-associated targets in global drug-target network of natural products. (A) KEGG pathway enrichment analysis; (B) Biological process enrichment analysis; (C) Molecular function enrichment analysis. [file Image1.TIF]
